# Absolute oral bioavailability and disposition kinetics of puerarin in female rats

**DOI:** 10.1186/s40360-018-0216-3

**Published:** 2018-05-25

**Authors:** Tosapol Anukunwithaya, Pilaslak Poo, Natthaphon Hunsakunachai, Ratchanee Rodsiri, Suchinda Malaivijitnond, Phisit Khemawoot

**Affiliations:** 1 Department of Pharmacology and Physiology, Faculty of Pharmaceutical Sciences, Chulalongkorn University, Bangkok, 10330 Thailand; 2 Department of Biology, Faculty of Science, Chulalongkorn University, Bangkok, 10330 Thailand; 3 Preclinical Pharmacokinetics and Interspecies Scaling for Drug Development Research Unit, Chulalongkorn University, Bangkok, 10330 Thailand

**Keywords:** Pharmacokinetics, Puerarin, Rats

## Abstract

**Background:**

*Pueraria candollei var*. *mirifica* is a medicinal plant that is promoted as a “Champion Product” by the Government of Thailand. This plant has been reported to relieve postmenopausal symptoms, prevent and reverse bone loss, inhibit the growth of breast cancer, and alleviate cardiovascular diseases in preclinical and clinical studies. However, there is little information on the oral bioavailability and tissue distribution of puerarin with respect to its pharmacodynamic activities. Therefore, the aim of this study was to determine the pharmacokinetics of puerarin, including absorption, distribution, metabolism, and elimination, in rats. Moreover, this is the first study to examine the tissue distribution of puerarin in the hippocampus, femur, tibia, and mammary gland.

**Methods:**

Adult female rats were administered puerarin at 1 mg/kg intravenously or 5 and 10 mg/kg orally. Blood, tissue, urine, and feces were collected and analyzed by liquid chromatography–tandem mass spectrometry.

**Results:**

Puerarin reached a maximum concentration in the blood of 140–230 μg/L within 1 h of oral dosing, and had an absolute oral bioavailability of approximately 7%. Following intravenous administration, puerarin was widely distributed in several tissues, including the hippocampus, heart, lung, stomach, liver, mammary gland, kidney, spleen, femur, and tibia. Approximately 50% of the intravenous dose was excreted as glucuronide metabolites via the urinary route.

**Conclusions:**

The absolute oral bioavailability of puerarin was approximately 7% at doses of 5 and 10 mg/kg. Puerarin was widely distributed to several organs related to the diseases of aging, including the hippocampus, femur, tibia, and mammary gland. Glucuronides were the major metabolites of puerarin and were mainly excreted in the urine. These results are useful for the development of puerarin and *Pueraria candollei var*. *mirifica* as phytopharmaceutical products.

## Background

**Fig. 1:**
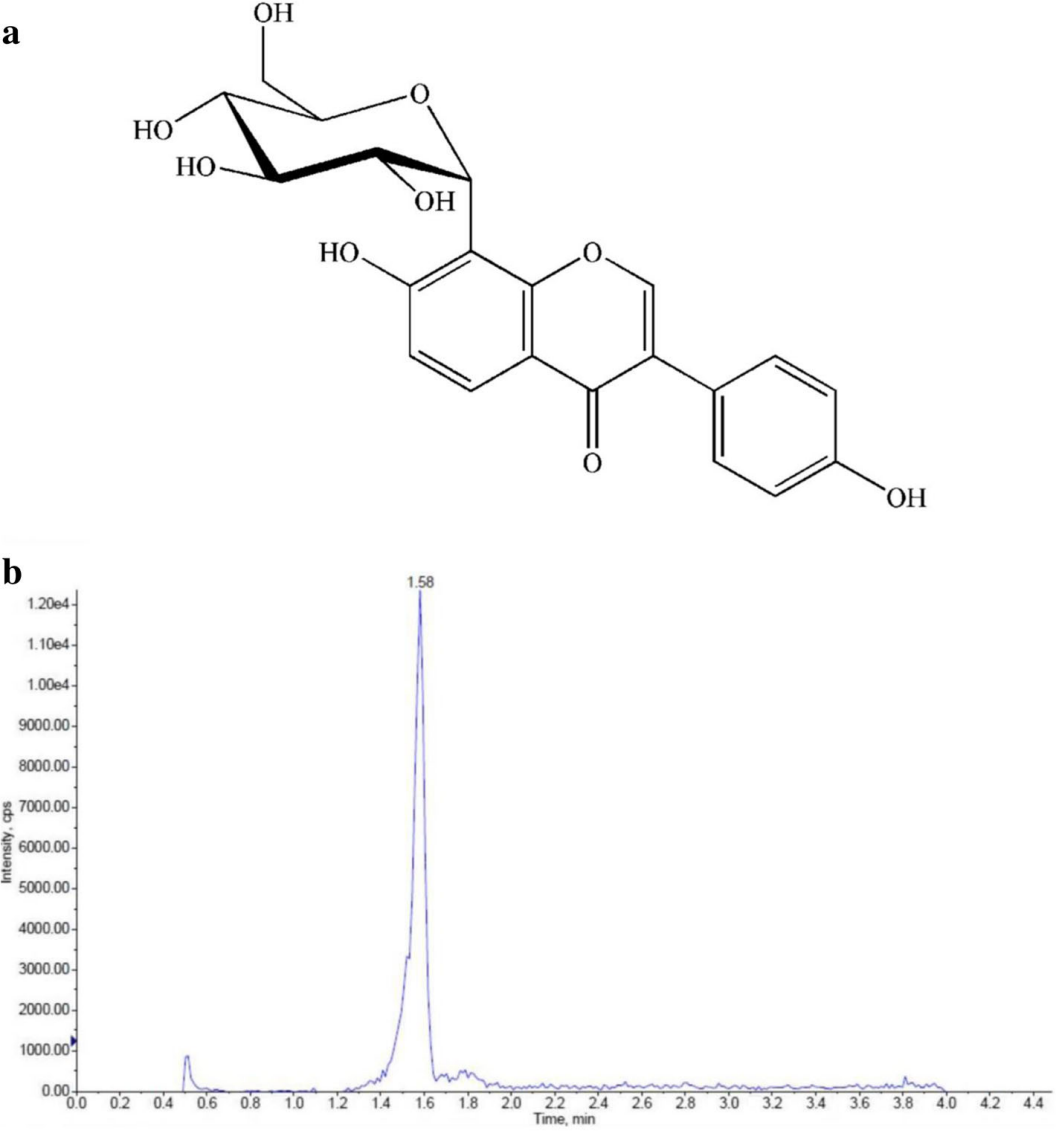
**a** Structure of puerarin. **b** LC-MS/MS chromatogram of 10 ng/mL puerarin spiked in rat plasma

Puerarin (7,4′-dihydroxy-8-C-glucosylisoflavone, Fig. 1a) is one of the major active phytoestrogens in *Pueraria candollei var*. *mirifica*, an endemic Thai plant of the family Leguminosae. This plant has been reported to relieve postmenopausal symptoms [1, 2], prevent and reverse bone loss [3–5], inhibit the growth of breast cancer [6], and alleviate cardiovascular diseases [7–9] in preclinical and clinical studies. There is also increasing evidence of a beneficial role of puerarin and *Pueraria candollei var*. *mirifica* in the prevention of and therapy for diseases of aging such as osteoporosis, neurodegeneration, diabetes, and cardiovascular disease [5, 8, 10, 11]. However, there is little information on the oral bioavailability and tissue distribution of puerarin, with respect to its pharmacodynamic activities.

Puerarin has an aqueous solubility of 0.46 mg/mL [12] and a partition coefficient of 1.95. In pharmacokinetics studies using oral dosing in animal models, puerarin reaches a maximum plasma concentration (C_max_) in 0.45–5.00 h and has an absorption half-life of 0.80–1.00 h after dosing [13–17]. Distribution of puerarin to the liver, spleen, kidney, lung, heart, and brain occurs within approximately 1 h following a single oral administration at 20 mg/kg. In the tested organs, C_max_ was reached at 2.5 h post-treatment [18]. Prasain et al. (2004) suggested that puerarin undergoes hepatic phase I metabolism via a cytochrome P450 enzyme and proposed that the metabolite was dihydroxylated puerarin [19]. However, Luo et al. reported that the major metabolic pathway of puerarin following intravenous administration is phase II glucuronidation to give puerarin-7-O-glucuronide and puerarin-4′-O-glucuronide, through glucuronidation at 7-OH and 4′-OH [20]. Excretion of puerarin has been proposed to occur mainly via the urinary system, with negligible amounts of puerarin and its glucuronide metabolites excreted via the hepatobiliary route [21].

The aim of this study was to determine the pharmacokinetics and absorption, distribution, metabolism, and elimination (ADME) properties of puerarin in the rat. The tissue distribution of puerarin in the hippocampus, femur and tibia, and mammary gland was investigated, based on the pharmacodynamic effects reported for neurodegenerative disease, osteoporosis, and breast cancer, respectively. The puerarin doses used in this study were based on estrogenic effects in the reproductive organs [22] and bone [23] in female rats. A liquid chromatography-tandem mass spectrometry (LC-MS/MS) method was developed to determine the puerarin level in biological samples following the administration of puerarin at 1 mg/kg intravenously (IV) and 5 and 10 mg/kg orally (PO) in healthy female rats.

## Methods

### Chemicals

Puerarin powder for the pharmacokinetic study was supplied by Dr. Suchinda Malaivijitnond, Department of Biology, Faculty of Science, Chulalongkorn University, Thailand. Glycyrrhetinic acid was purchased from Wako Pure Chemical Industries, Japan. β-Glucuronidase from *Escherichia coli* (Type VII-A) was purchased from Sigma-Aldrich (St. Louis, MO, USA). Isoflurane was purchased from Minrad International, Inc. (Orchard Park, NY, USA). Puerarin (analytical grade) was purchased from Pure Chemistry Scientific, Inc. (Burlington, MA, USA). Dimethyl sulfoxide (DMSO) was purchased from Sigma-Aldrich.

### Animals

Twenty-eight female Sprague-Dawley rats aged 12 weeks old were obtained from the National Laboratory Animal Centre, Mahidol University, Thailand. The animals were housed for 4 weeks at 25 ± 2 °C under a 12-h light/dark cycle with free access to food and water. The rats were moved to a metabolic cage one day before the experiment and kept in this cage for 72 h after the experiment. Rats weighing 300–400 g were used in pharmacokinetic studies. The sample size was four animals per experiment, based on OECD Guidelines for the Testing of Chemicals [24] and USFDA Guidance for Bioavailability and Bioequivalence Studies [25]. Experiments complied with the animal protocol guidelines of the Faculty of Pharmaceutical Sciences, Chulalongkorn University (approval number: 15–33-001, approval date: April 22, 2015).

### Pharmacokinetic studies

Twelve rats were divided into three groups of four animals each for the administration of puerarin at the doses of 1 mg/kg IV, and 5 and 10 mg/kg PO. The puerarin solution for administration to animals was prepared in 20% DMSO in normal saline. Rats were anaesthetized with isoflurane by the chamber induction method before IV administration and blood collection to reduce pain and injury. Blood samples (300 μL) taken from the lateral tail vein at 0, 0.083 (5 min), 0.25, 0.50, 1, 2, 4, 8, 12, and 24 h after administration were collected in heparinized tubes, centrifuged at 1500×*g* for 10 min, and kept at − 20 °C for further analysis. Urine and feces were collected in three periods, 0–24, 24–48, and 48–72 h, following IV and PO administration and stored at − 20 °C.

To examine the tissue distribution of puerarin, another 16 rats received IV administration of 1 mg/kg puerarin and were subsequently sacrificed by cervical dislocation at 5 min and 1, 2, and 4 h after administration. Major organs including the brain (particularly hippocampus), liver, kidney, spleen, stomach, heart, lung, small intestine, mammary gland, femur, and tibia were collected, rinsed with ice-cold saline, and stored at − 20 °C until analysis.

Determination of plasma creatinine, aspartate transminase (AST), and alanine transaminase (ALT) levels at 0 and 24 h after dosing were made by the Professional Laboratory Management Corp Co., Ltd. (accredited ISO 15189) with the automated analyzer Cobas® 6000 (F. Hoffmann-La Roche, Ltd., Basel, Switzerland) using an enzymatic method (chemiluminescence) for creatinine quantitation and a kinetic method (according to the International Federation of Clinical Chemistry and Laboratory Medicine recommendations) for AST and ALT quantification to evaluate pre- and post-dose kidney and liver function.

### Sample preparation

All biological samples were extracted by the protein precipitation method as previously described [26], with minor modifications. Briefly, 50 μL of plasma and urine samples were mixed directly with 200 μL of methanol containing 10 ng of glycyrrhetinic acid as the internal standard, whereas 50 mg of tissue and feces samples were chopped into small pieces, homogenized, and mixed in 200 μL methanol containing the internal standard. Then, samples were centrifuged for 10 min at 5000×*g* to precipitate proteins. A volume of 150 μL of the supernatant was transferred into a sample vial and 10 μL of the supernatant was injected into the LC-MS/MS system. For the femur and tibia, decalcified bones were prepared by soaking in 10% EDTA solution for 2 weeks, and the bones were chopped and processed in a similar manner to the other tissues [27].

For the determination of glucuronide conjugates of puerarin, 50 μL of plasma or urine was added to 50 μL of 0.1 M potassium phosphate buffer (pH 6.8) containing 1500 units of β-glucuronidase. The mixture was incubated at 37 °C for 15 min, and the reaction was stopped by adding 400 μL of methanol containing 20 ng of glycyrrhetinic acid as the internal standard. The mixture was centrifuged at 5000×*g* for 10 min, the supernatant was collected, and 10 μL of the supernatant was injected into the LC-MS/MS system. For solid samples, 50 mg of tissue and feces were chopped into small pieces, homogenized, and mixed in 50 μL of 0.1 M potassium phosphate buffer (pH 6.8) containing 1500 units of β-glucuronidase. These mixtures were then processed in a similar manner to the plasma and urine mixtures.

### LC-MS/MS analysis

The analytical method was performed according to Li et al. [13] and Prasain et al. [19], with minor modifications to allow for the analysis of puerarin with good linearity, precision, and high accuracy. An Ultra LC 100 (Eksigent, Canada) system was equipped with a Synergi Fusion-RP C18 column as the stationary phase (Phenomenex, Torrance, CA, USA). The LC system used 100% methanol and 0.2% formic acid in water (pH 2.5) with a flow rate of 0.5 mL/min. The mobile phase was rinsed with 10% methanol for 0.5 min, increased to 90% methanol from 1.5 to 3.5 min, and then decreased to 10% methanol from 4 to 4.5 min. The retention time of puerarin was 1.58 min, and that of the internal standard was 2.09 min. Detection was conducted in negative ionization mode by monitoring precursor ion to product ion transitions with mass to charge ratios of 415/295 (puerarin) and 469/409 (glycyrrhetinic acid). The chromatograms were essentially free from endogenous interference (Fig. 1b). The limit of detection was estimated to be 0.16 μg/L, with a signal-to-noise ratio of 5. Calibration curves were constructed by the analysis of puerarin at 200, 100, 50, 25, 12.5, 6.25, 3.12, and 1.56 μg/L, with good correlation coefficients (R^2^ > 0.99) for all matrices.

### Data analysis

Pharmacokinetic parameters were calculated by non-compartmental analysis using PK Solutions 2.0 software (Summit Research Services, Montrose, CO, USA). The acquired results are reported as the mean ± standard deviation with a significance level of 0.05. Statistical analysis was performed by non-parametric tests using SPSS ver. 16 (SPSS, Chicago, IL, USA). The following pharmacokinetic parameters are reported: maximum plasma concentration (C_max_), time to maximum plasma concentration (T_max_), area under the plasma concentration-time curve from zero to the last observed time (AUC_0–t_) or infinity (AUC_0–∞_), volume of distribution (Vd), total clearance (CL), mean residence time (MRT), and elimination half-life (T_1/2_). The absolute oral bioavailability of puerarin was calculated as (AUC_po_/dose_po_) divided by (AUC_iv_/dose_iv_). The tissue-to-plasma ratio of puerarin was calculated from the tissue concentration divided by the plasma concentration in each rat at the same time point. The percentage recovery of puerarin was calculated by dividing the puerarin level found in urine or feces by the administered dose.

## Results

### Animal tolerability

Female rats that received puerarin at 1 mg/kg IV or 5 or 10 mg/kg PO had a normal physiological and morphological appearance pre-dose and 24 h post-dose (Table 1). Liver biochemical profiles showed no significant changes in AST and ALT levels pre-dose and 24 h post-dose in all rats, although slight decreases in AST and ALT were observed at 24 h. In the kidney biochemical profiles, blood creatinine showed no significant changes from pre-dose to post-dose following IV or PO administration of puerarin.

**Table 1:** Physical appearance and plasma biochemical profiles before (0 h) and 24 h after administration of puerarin at 1 mg/kg IV and 5 and 10 mg/kg PO

Parameters	Administration of Puerarin					
	1 mg/kg IV		5 mg/kg PO		10 mg/kg PO	
	0 h	24 h	0 h	24 h	0 h	24 h
Physical appearance	Normal	Normal	Normal	Normal	Normal	Normal
AST (U/L)	61.67 ± 9.36	55.83 ± 2.90	57.67 ± 5.10	56.00 ± 6.84	55.60 ± 7.96	57.40 ± 5.12
ALT (U/L)	27.00 ± 26.18	11.20 ± 16.22	26.33 ± 18.78	13.17 ± 17.40	25.00 ± 13.92	12.17 ± 9.76
Creatinine (mg/dL)	0.20 ± 0.00	0.20 ± 0.00	0.21 ± 0.02	0.21 ± 0.02	0.21 ± 0.10	0.21 ± 0.08

Data are presented as mean ± S.D. (*n* = 4). ^*^p < 0.05, 0 h vs. 24 h

### Plasma concentration-time profile and oral bioavailability

**Fig. 2:**
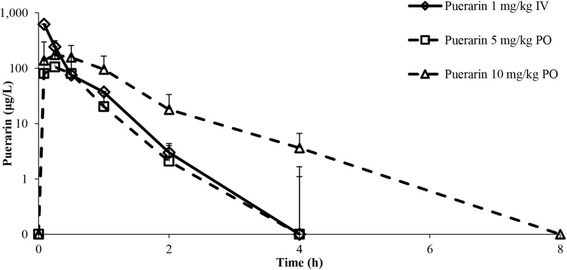
Plasma concentration-time profiles of puerarin after administration of puerarin at 1 mg/kg IV (◊), 5 mg/kg PO (□) and 10 mg/kg PO (∆). Data are shown as mean ± S.D. (*n* = 4)

Mean plasma concentration-time profiles of puerarin following IV and PO administration in rats are shown in Fig. 2. Pharmacokinetic parameters of puerarin from non-compartmental analysis are summarized in Table 2. Following IV administration, the concentration declined rapidly and puerarin was cleared from the systemic circulation within 4 h, with C_max_, 621.96 ± 170.72 μg/L; AUC_0–∞_, 292.23 ± 108.93 μg.h/L; Vd, 1.16 ± 0.56 L/kg; CL, 5.43 ± 2.66 L/h/kg; MRT, 0.24 ± 0.06 h; and T_1/2_, 0.21 ± 0.06 h. Following PO administration of 5 and 10 mg/kg, the maximum concentration was reached within 1 h, and puerarin decreased to below the detection limit at 4–8 h post-dosing. C_max_ ranged from 140 to 230 μg/L and AUC_0-∞_ was 110–210 μg.h/L. The T_1/2_ of puerarin ranged from 0.86–0.88 h, with no significant difference between doses. The absolute oral bioavailability of puerarin was approximately 7% at doses of 5 and 10 mg/kg.

**Table 2:** Pharmacokinetic parameters of puerarin and puerarin glucuronide after administration of puerarin at 1 mg/kg IV and 5 and 10 mg/kg PO

Parameters	Administration of Puerarin		
	1 mg/kg IV	5 mg/kg PO	10 mg/kg PO
Puerarin			
C_max_ (μg/L)	621.96 ± 170.72	145.47 ± 84.11	228.00 ± 164.84
T_max_ (h)	N/A	0.19 ± 0.09	0.33 ± 0.21
AUC_0-t_ (μg.h/L)	291.47 ± 108.05	109.45 ± 60.61	204.57 ± 157.47
AUC_0-∞_ (μg.h/L)	292.23 ± 108.93	109.58 ± 60.55	212.20 ± 157.50
Vd (L/kg)	1.16 ± 0.56	101.59 ± 119.57	90.17 ± 18.28
MRT (h)	0.24 ± 0.06	0.67 ± 0.21	0.88 ± 0.20
T_1/2_ (h)	0.21 ± 0.06	0.88 ± 0.49	0.86 ± 56.31
CL (L/h/kg)	5.43 ± 2.66	65.06 ± 52.75	74.79 ± 0.17
Absolute bioavailability (%)	100	7.50	7.29
Puerarin glucuronide			
AUC_0-t_ (μg.h/L)	2133.20	73.30	7207.40
AUC_puerarin glucuronide_ / AUC_puerarin_	7.35	0.67	34.65

C_max_, maximum concentration, T_max_, time to reach maximum concentration, AUC_0-t_, area under concentration-time curve from time 0 to last observed time, AUC_0-∞_, area under concentration-time curve from time 0 to infinity, Vd, apparent volume of distribution, MRT, mean resident time, T_1/2,_ half-life, CL, apparent clearance, N/A, not available

Data are presented as mean ± S.D. (*n* = 4). *p < 0.05 between oral doses

### Tissue distribution

**Fig. 3:**
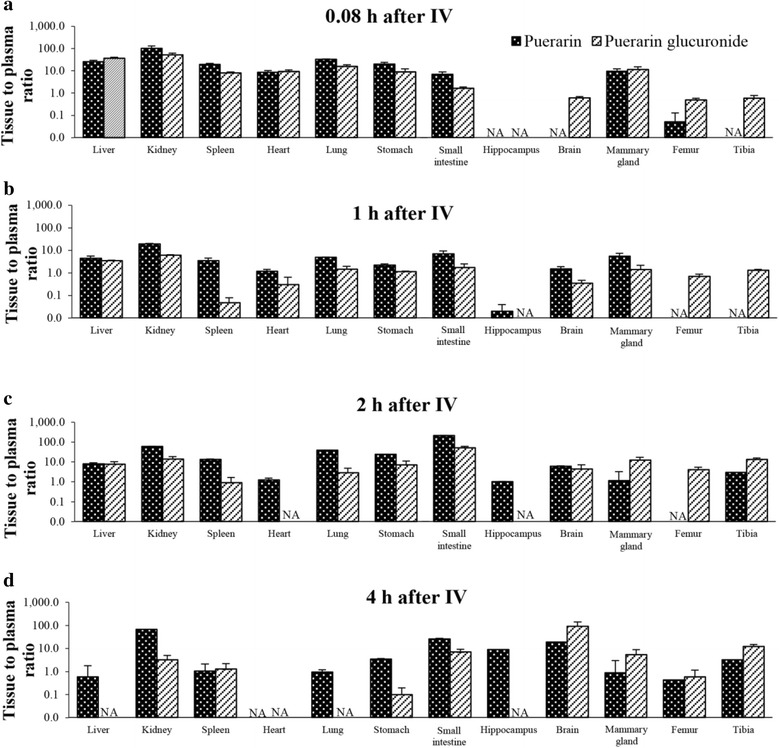
Tissue-to-plasma ratios of puerarin (black bars) and puerarin glucuronide (grey bars) in internal organs at (**a**) 5 min, (**b**) 1.0 h, (**c**) 2.0 h and (**d**) 4.0 h after administration of puerarin at 1 mg/kg IV. Data are shown as mean ± S.D. (*n* = 4), NA = below limit of detection

Tissue-to-plasma ratios of puerarin in rats at 5 min and 1, 2, and 4 h after administration of puerarin at 1 mg/kg IV are shown in Fig. 3. At 5 min, the highest levels of puerarin were found in the kidney, followed by the lung, stomach, liver, mammary gland, and small intestine, all of which are highly perfused organs. The ratios in these organs were 10–100 at 5 min after IV dosing, and decreased to 1–10 at 1 h after dosing. At 2 h, puerarin was detected in most organs, including the hippocampus, heart, lung, stomach, liver, mammary gland, kidney, spleen, and tibia, but not in the femur. The ratios in most organs continued to decrease up to 4 h, but there was an increase in poorly perfused tissue such as bone. Interestingly, the tissue-to-plasma ratio of puerarin in the hippocampus and brain increased significantly and continuously from 5 min to 1, 2, and 4 h after dosing.

### Metabolism

**Fig. 4:**
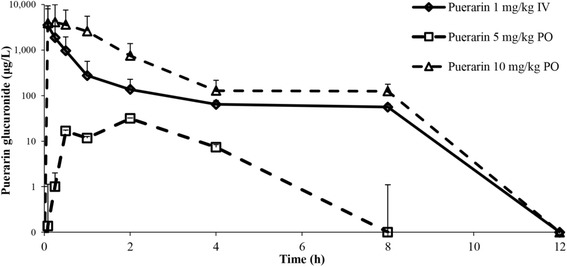
Plasma concentration-time profiles of puerarin glucuronide after administration of puerarin at 1 mg/kg IV (◊), 5 mg/kg PO (□) and 10 mg/kg PO (∆). Data are shown as mean ± S.D. (*n* = 4)

Mean plasma concentration-time profiles for puerarin glucuronide after administration of puerarin in rats are shown in Fig. 4. The AUC_puerarin glucuronide_/AUC_puerarin_ ratio was approximately 7 after IV dosing. The concentration of puerarin glucuronide was higher than unchanged puerarin by about 30 times following PO administration of 10 mg/kg puerarin. Regarding the tissue distribution, a high ratio of glucuronide metabolites was found in most tissues at 5 min after IV dosing. The tissue-to-plasma ratio of glucuronide metabolites decreased over time in most tissues, except for the brain and bone. However, the ratios of unchanged puerarin and puerarin glucuronide in the mammary gland remained at comparable levels for 4 h after dosing.

### Excretion

The percentage recovery of unchanged puerarin and puerarin glucuronide in rat urine or feces after administration are shown in Table 3. Unchanged puerarin was present in urine and feces at less than 1% following PO and IV dosing. In the IV group, puerarin glucuronide was mainly excreted in urine in the first 24 h; this accounted for almost 50% of the administered dose. Approximately 15% of puerarin glucuronide was excreted in the feces within 72 h after IV dosing. At 10 mg/kg PO, approximately 10% of puerarin glucuronide was found in the urine in the 0–24 h period. A negligible amount of puerarin glucuronide was found in feces 0–72 h after PO dosing.

**Table 3:** Percent recovery of unchanged puerarin and puerarin glucuronide after administration of puerarin at 1 mg/kg IV and 5 and 10 mg/kg PO

Recovery (%)	Administration of Puerarin		
	1 mg/kg IV	5 mg/kg PO	10 mg/kg PO
Puerarin			
Urine_0-24h_	< 1.00	< 1.00	< 1.00
Urine_24-48h_	< 1.00	< 1.00	< 1.00
Urine_48-72h_	< 1.00	< 1.00	< 1.00
Faeces_0-24h_	< 1.00	< 1.00	< 1.00
Faeces_24-48h_	< 1.00	< 1.00	< 1.00
Faeces_48-72h_	< 1.00	< 1.00	< 1.00
Puerarin glucuronide			
Urine_0-24h_	47.53 ± 51.37	< 1.00	12.13 ± 13.01
Urine_24-48h_	2.38 ± 0.97^*^	< 1.00	< 1.00
Urine_48-72h_	1.89 ± 0.45^*^	< 1.00	< 1.00
Faeces_0-24h_	7.66 ± 2.51^*^	< 1.00	1.94 ± 2.16
Faeces_24-48h_	3.55 ± 0.55^*^	< 1.00	< 1.00
Faeces_48-72h_	3.45 ± 0.80^*^	< 1.00	< 1.00

Data are presented as mean ± S.D. (*n* = 4). *p < 0.05 among doses

## Discussion

Puerarin has therapeutic effects on the cardiovascular and cerebrovascular systems and on bone [8]. Further understanding of these pharmacological activities requires information on the oral bioavailability and tissue distribution of puerarin. Therefore, this study was conducted to evaluate the ADME properties of puerarin at pharmacologically active sites in female rats. The rats could tolerate puerarin at 1 mg/kg IV and 5 and 10 mg/kg PO, which are pharmacologically active doses. All rats had a normal appearance without signs of toxicity after dosing. In addition, there were no significant changes in liver and kidney biomarkers within 24 h after dosing (Table 1). This suggests that puerarin is safe in rats in the pharmacologically active dose range, consistent with the findings of Chung et al. [28], in which male and female rats fed with puerarin for 28 days showed no toxicity at a dose up to 250 mg/kg per day.

The C_max_ of puerarin was reached within 5 min after PO dosing, and systemic clearance occurred until 8–12 h (Fig. 2). Previous studies of PO administration of a puerarin suspension resulted in C_max_ at 0.45–5.00 h [12–16]. The wider range of T_max_ in these studies might be due to the use of a puerarin suspension formulation. The puerarin used in our study was prepared as a clear solution that was readily absorbed, resulting in a shorter T_max_ compared to that for the suspension. Thus, we conclude that puerarin is absorbed rapidly from the gastrointestinal (GI) tract. Biotransformation of puerarin via the formation of glucuronide conjugates was also clearly observed within the first 5 min, indicating that the metabolism of puerarin through the glucuronidation pathway also occurs rapidly. The primary difference between puerarin glucuronides of the two oral doses could be due to the activity of distinct metabolic pathways at different doses of puerarin. At a low dose, puerarin could be metabolized via multiple metabolic pathways, e.g., sulfation and/or glucuronidation. Therefore, the level of puerarin glucuronide appeared to be low at a low dose. However, a high dose of puerarin could saturate minor metabolic pathways such as sulfation. At this point, most of the puerarin molecules might be biotransformed via a major metabolic pathway or glucuronidation, and thus the level of puerarin glucuronide was higher with a higher dose.

The amounts of plasma puerarin and glucuronide metabolites were investigated as a function of the oral dose to examine dose proportionality. The plasma concentration of puerarin increased linearly from 5 to 10 mg/kg PO, but at a higher dose of 20–100 mg/kg, the oral bioavailability of puerarin was less than 1% (data not shown). This may be because at the higher dose, puerarin is incompletely dissolved due to precipitation in vivo. To maintain a consistent dose volume of 1 mL/kg, we varied the concentration of puerarin from 5 to 100 mg/mL in 20% DMSO in normal saline. Puerarin is in class IV of the Biopharmaceutical Classification System, indicating low solubility and low permeability, and is a weak acid that is ionized at higher pH. Thus, it will have lower solubility in the acid medium in the stomach of rats, which may cause precipitation [29]. Therefore, the lower bioavailability of puerarin at a higher oral dose may be strongly affected by the dissolution state of puerarin in GI fluid. Neither puerarin nor puerarin glucuronide were found in plasma or urine following oral dosing at 20–100 mg/kg. The low oral bioavailability of lead compounds from natural resources is a common phenomenon in herbal pharmacokinetics. Low percentage in oral bioavailability might be improved when administered in the form of crude extract or when combined with other active ingredients.

To the best of our knowledge, this is the first study on the tissue distribution of puerarin and puerarin glucuronide in the hippocampus, femur, tibia, and mammary gland (Fig. 3). Puerarin was widely distributed in several organs, and especially those with high porosity, consistent with its pharmacodynamic activities in these organs [19]. In addition, the volume of distribution of puerarin after IV dosing was approximately 1.16 L/kg, which implies a good tissue distribution. This correlates well with the physiochemical properties of puerarin including high lipophilicity (XlogP 1.95) and low water solubility (0.46 mg/mL). The tissue distribution in the current study showed that puerarin at 1 mg/kg IV reaches appropriate levels for pharmacodynamic activities in the hippocampus, femur, tibia, and mammary gland, which are related to the prevention of and therapy for neurodegenerative diseases and osteoporosis [5, 8, 10, 11]. This tissue distribution in rats correlates well with the use of puerarin in traditional medicine. The test compound could be distributed into several organs including the hippocampus, bone, and mammary gland. However, there are some discrepancies in rat and human physiology, e.g., tissue barriers, tissue blood flow, and drug metabolizing enzymes. Extrapolation from rat pharmacokinetics to human usage will likely require further investigation and careful interpretation.

Glucuronide metabolites of puerarin were also detected in most tissues. The AUC_puerarin glucuronide_/AUC_puerarin_ ratios were 7 after puerarin IV dosing and 30 after puerarin at 10 mg/kg PO (Table 2). Glucuronide metabolites found in biological samples after puerarin administration are formed by UDP glucuronosyltransferases [18]. We found that puerarin was biotransformed to a glucuronide within 5 min in plasma (Fig. 4). These results indicate that glucuronidation occurs rapidly and that most puerarin is converted into glucuronides. This first pass metabolism could reduce the amount of puerarin in the systemic circulation and may account for the relatively low oral bioavailability of puerarin. Sex and interspecies differences of the enzymes responsible for puerarin biotransformation need to be explored for phytopharmaceutical product development [30–32].

The percentage of unchanged puerarin in urine and feces was less than 1% over 72 h after IV or PO dosing, indicating that puerarin is biotransformed to glucuronide metabolites before excretion via the bile or urine. We detected puerarin glucuronide in urine and feces using enzymatic hydrolysis of glucuronidase under optimized conditions. Puerarin was mainly excreted in the form of puerarin glucuronide, and approximately 50% of the administered dose of 1 mg/kg IV was detected in the urine as the glucuronide during the first 24 h. After PO dosing, 1–10% of the administered dose was excreted in urine as the glucuronide. The difference in the percentage recovery between the different routes of administration (IV or PO) might be explained by distinct metabolic pathways. Prasain et al. [19] reported that puerarin can be hydrolyzed to daidzein by microbial metabolism in the GI tract, and then reduced to dihydrodaizein and equol after oral dosing. In this study, we also detected daidzein and equol as minor metabolites in urine and feces at 72 h after dosing. However, the levels of daidzein and equol in urine and feces accounted for only 1–2% of the administered dose (data not shown).

## Conclusions

This study showed that puerarin has an oral bioavailability of approximately 7%. For tissue distribution following IV administration, puerarin was widely distributed to several organs including the hippocampus, femur, tibia, and mammary gland. Glucuronides were the major metabolites of puerarin and were mainly excreted in the urine. Further studies in comparative pharmacokinetics of puerarin when administered as a standardized extract compared with pure compound will be required for the phytopharmaceutical product development of *Pueraria mirifica*.
